# Compact source for quadripartite deterministically entangled optical fields

**DOI:** 10.1016/j.fmre.2022.11.006

**Published:** 2022-11-23

**Authors:** Yanhong Liu, Yaoyao Zhou, Liang Wu, Jiliang Qin, Zhihui Yan, Xiaojun Jia

**Affiliations:** a Department of Physics, Taiyuan Normal University, Jinzhong 030619, China; b Institute of Computational and Applied Physics, Taiyuan Normal University, Jinzhong 030619, China; c State Key Laboratory of Quantum Optics and Quantum Optics Devices, Institute of Opto-Electronics, Shanxi University, Taiyuan 030006, China; d Collaborative Innovation Center of Extreme Optics, Shanxi University, Taiyuan 030006, China; e College of Information Engineering, Shanxi Vocational University of Engineering Science and Technology, Jinzhong 030619, China

**Keywords:** Quadripartite entanglement, Single NOPA, Compact source, GHZ state, Cluster state

## Abstract

Since entangled multiple optical fields were identified as the building blocks of quantum networks, the quadripartite entangled optical fields have been produced by using four degenerate optical parametric amplifiers or two nondegenerate optical parametric amplifiers (NOPAs). However, realizing an efficient and compact source for multiple quantum users has remained an outstanding challenge, hindering their practical applications. Here, we proposed a compact and feasible scheme to deterministically entangle four spatially separated optical fields, employing only a single NOPA. Accordingly, two-sided output NOPA-based optical fields were coupled on a beam splitter network to form the quadripartite entangled state, causing the deterministic generation of both the Greenberger–Horne–Zeilinger (GHZ) and the linear cluster states in this compact entanglement source. We also obtained the optimal experimental parameters based on the simulation results, thereby providing a direct reference for experimental implementation. Our findings propose that the resultant GHZ and linear cluster states can be potentially applied in quantum-enhanced information science, specifically in quantum secret sharing, controlled quantum teleportation networks, and quantum-entangled atomic ensemble networks.

## Graphical abstract

**Figure:**
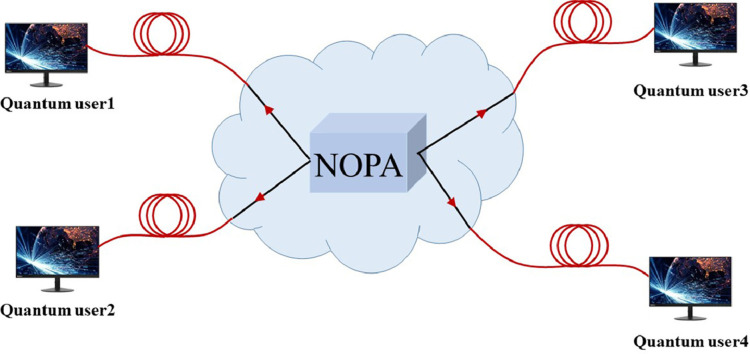

## 1 Introduction

Quantum entanglement is a valuable resource in quantum information science, such as quantum computation [1], [2], [3], [4], quantum communication [5], [6], [7], [8], [9], [10], [11], [12], [13], and quantum metrology [14], [15], [16], [17], [18], [19], and the multipartite entangled optical fields are a core resource. In quantum information science, a quantum network enables the processing and transmission of quantum information across the network, which requires multipartite entangled states [20], [21], [22], [23]. While light is the ideal carrier of quantum information, having the advantages of fast speed and good coherence in a large-scale quantum network, the development of quantum networks forms a building block for the entanglement of multiple spatially separated optical fields. Preparations of large-scale entangled optical fields have been realized in the time-domain [24], [25], [26], frequency-domain [27], [28], and spatial-domain [29], respectively. These quantum networks potentially enable quantum communication [11], distributed quantum sensing and quantum networks [16], [30], [31].

While discrete-variable quantum information [32], [33], [34], [35] realizes probabilistic entanglement among ten photons [36], the continuous-variable (CV) quantum information approach enables the deterministic generation, manipulation, and measurement of quantum states [37], [38], [39]. While there are many CV multipartite entangled states such as Greenberger–Horne–Zeilinger (GHZ) [40], [41], [42] and cluster states [43], [44], [45], which enable potential utilities in a quantum network, the optical parametric amplifier is one of the most widely used devices to generate nonclassical light states [46], [47]. These CV multipartite entangled states are generated by coupling multiple squeezed optical fields on a beam splitter network [48], [49]. Among these states are the four degenerate optical parametric amplifiers (DOPAs) that produce four squeezed optical fields, which are then coupled on a beam splitter network to form a quadripartite entangled state [45]; however, the nondegenerate optical parametric amplifier (NOPA) can reduce the cost of quantum resources, causing the quadripartite entangled state to be produced with only two NOPAs and a beam splitter network [44]. Still, with the development of quantum information, the deterministic generation of entanglement among multiple optical fields using a compact and efficient way is a key to the practical applications of a large-scale quantum network.

Here, we proposed a compact and feasible scheme to deterministically generate quadripartite entangled optical fields. In our scheme, only one NOPA, comprising two output ports and operating at a parametric de-amplification status, and three optical beam splitters are needed. As a result, the down-conversion optical fields produced from the type-II phase-matching nonlinear processing using these two output ports were in orthogonal polarization states, which could then be separated conveniently by using half-wave plates and polarization beam splitters (PBSs). Notably, the output optical fields from these two PBSs were two quadrature phase and two quadrature amplitude squeezed states, respectively [44]. Next, these four quadrature squeezed optical fields were coupled on a beam splitter network to form four spatially separated entangled optical fields. Investigations revealed that this compact entanglement source flexibly generated both the GHZ and the linear cluster states by controlling the phase of the controlled beam splitter (CBS). Then, both of them were verified by the inseparability criteria of multipartite entanglement, followed by an analysis of the performance of quadripartite entangled states. Consequently, optimal experimental parameters were obtained, providing a direct reference for experimental implementation.

## 2 Theoretical model

**Fig. 1:**
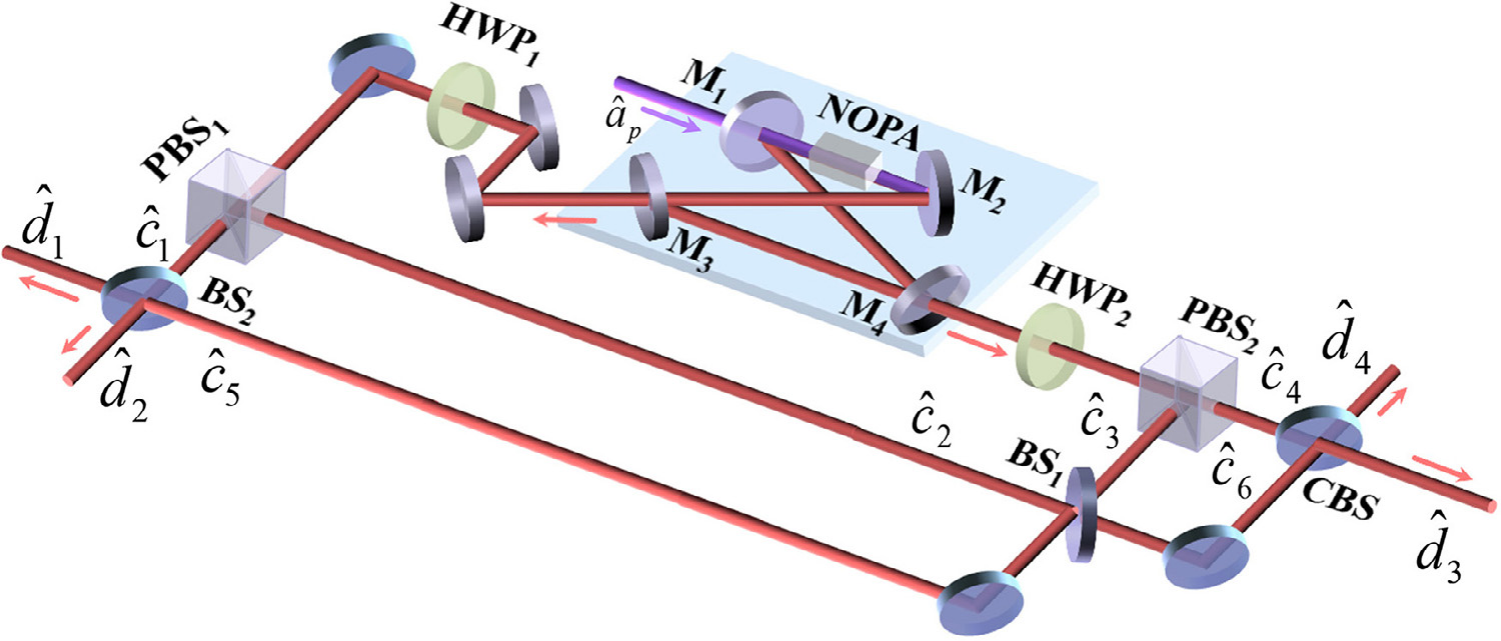
**Schematic showing of the compact source for quadripartite deterministic entanglement.** NOPA, nondegenerate optical parametric amplifier; HWP${}_{1,2}$, half-wave plate; PBS${}_{1,2}$, polarizing beam splitter; BS${}_{1,2}$, beam splitter; CBS, controlled beam splitter. ${\hat{c}}_{1}$, ${\hat{c}}_{4}$, and ${\hat{c}}_{2}$, ${\hat{c}}_{3}$ comprise the quadrature phase and quadrature amplitude squeezed states, respectively; ${\hat{d}}_{1}$, ${\hat{d}}_{2}$, ${\hat{d}}_{3}$, and ${\hat{d}}_{4}$ are the output optical fields from the compact source.

In quantum optics, the optical fields are described by annihilative and creative operators $\hat{a}$ and ${\hat{a}}^{†}$, comprising the amplitude and phase quadratures, $\hat{X}$ and $\hat{Y}$, that correspond to the real and imaginary parts of the annihilation operator $\hat{a}$, as $\hat{X}=\hat{a}+{\hat{a}}^{†}$, $\hat{Y}=i\left({\hat{a}}^{†}-\hat{a}\right)$. The schematic depicting a compact source for quadripartite deterministic entanglement is shown in Fig. 1. In this protocol, the NOPA with two output ports is operating at a parametric de-amplification status, with each output port producing two fields with orthogonal polarization, which are separated by half-wave plate and PBS. These output optical fields from the two PBSs comprise two quadrature phase and two quadrature amplitude squeezed states, respectively. Specifically, the two amplitude quadrature squeezed optical fields were interfered at the first beam splitter $BS_{1}$ ($T_{0}^{\prime }/R_{0}^{\prime }$), with one comprising the output fields of $BS_{1}$ and a one phase quadrature squeezed optical field that were coupled on the second beam splitter $BS_{2}$ ($T_{1}^{\prime }/R_{1}^{\prime }$), and the other output field of the $BS_{1}$ and the other phase quadrature squeezed optical field were coupled on the CBS ($T_{2}^{\prime }/R_{2}^{\prime }$). Consequently, the final output optical fields from $BS_{2}$ and CBS formed quadripartite entangled states.

The NOPA designed in this study employed the four-mirror ring cavity configuration, which consisted of two spherical mirrors ($M_{1}$ and $M_{2}$), two flat mirrors ($M_{3}$ and $M_{4}$), and a type-II phase-matching crystal. Notably, we used one of the spherical mirrors $M_{1}$ as the input coupler, coating it with an antireflection for the pump field. As a result, the pump field ${\hat{a}}_{p}$ could be coupled into the cavity through an input coupler $M_{1}$, after which the other three cavity mirrors were coated with a highly reflective material for the pump field. Next, we mounted the spherical mirror $M_{2}$ on a piezoelectric transducer for scanning or locking the cavity length, causing the signal and idler fields to resonate with the cavity field; however, flat mirrors $M_{3}$ and $M_{4}$ were used as output couplers, ${\hat{a}}_{s_{1}}^{in}$ (${\hat{a}}_{i_{1}}^{in}$) and ${\hat{a}}_{s_{2}}^{in}$ (${\hat{a}}_{i_{2}}^{in}$) were the input signal (idler) fields through the output couplers $M_{3}$ and $M_{4}$ with the coupling rates to the cavity of the signal and idler fields $\gamma_{1}$ and $\gamma_{2}$ ($\gamma_{1}=T_{1}/2$ and $\gamma_{2}=T_{2}/2$, where $T_{1}$ and $T_{2}$ are the transmissivities of the couplers $M_{3}$ and $M_{4}$), respectively. Unfortunately, the intracavity loss L is unavoidable in real experiments as a result of imperfect optical components, causing the corresponding decay rate of intracavity loss to be $\gamma_{3}$ ($\gamma_{3}=L/2$), which introduces vacuum noise ${\hat{b}}_{1}^{in}$ and ${\hat{b}}_{2}^{in}$. Notwithstanding, we obtained the quantum Langevin motion equations for the parametric down-conversion optical fields (${\hat{a}}_{s}$ and ${\hat{a}}_{i}$) in a two-sided cavity and when the NOPA operates at parametric de-amplification, as follows:

(1) $$\begin{array}{ccc} \tau \frac{d{\hat{a}}_{s}}{dt} & = & -\kappa {\hat{a}}_{p}{\hat{a}}_{i}^{†}-\left(\gamma_{1}+\gamma_{2}+\gamma_{3}\right){\hat{a}}_{s}+\sqrt{2\gamma_{1}}{\hat{a}}_{s_{1}}^{in}+\sqrt{2\gamma_{2}}{\hat{a}}_{s_{2}}^{in}+\sqrt{2\gamma_{3}}{\hat{b}}_{1}^{in}\\ \tau \frac{d{\hat{a}}_{i}}{dt} & = & -\kappa {\hat{a}}_{p}{\hat{a}}_{s}^{†}-\left(\gamma_{1}+\gamma_{2}+\gamma_{3}\right){\hat{a}}_{i}+\sqrt{2\gamma_{1}}{\hat{a}}_{i_{1}}^{in}+\sqrt{2\gamma_{2}}{\hat{a}}_{i_{2}}^{in}+\sqrt{2\gamma_{3}}{\hat{b}}_{2}^{in} \end{array}$$

where τ=l/c is the round trip time of the optical field inside the NOPA, κ is the parametric coupling constant of the crystal, and $\gamma =\gamma_{1}+\gamma_{2}+\gamma_{3}$.

According to the operator linearization approach, operators are considered as the sum of the mean values $\bar{x}$ ($\bar{y}$) and the fluctuations $\delta \hat{X}$ ($\delta \hat{Y}$), $\hat{X}=\bar{x}+\delta \hat{X}$, $\hat{Y}=\bar{y}+\delta \hat{Y}$. In this approach, since the input signal and idler fields were considered vacuum states, their fluctuations were normalized: $\delta^{2}\hat{X}\left(a_{s_{1(2)}}^{in}\right)=\delta^{2}\hat{X}\left(a_{i_{1(2)}}^{in}\right)=\delta^{2}\hat{X}\left(b_{1(2)}^{in}\right)=1$, $\delta^{2}\hat{Y}\left(a_{s_{1(2)}}^{in}\right)=\delta^{2}\hat{Y}\left(a_{i_{1(2)}}^{in}\right)=\delta^{2}\hat{Y}\left(b_{1(2)}^{in}\right)=1$. By solving the above quantum Langevin motion equations and combining with the input–output relationship of the signal and idler optical fields $\delta {\hat{a}}_{s_{1}\left(i_{1}\right)}^{out}=\sqrt{2\gamma_{1}}\delta {\hat{a}}_{s(i)}-\delta {\hat{a}}_{s_{1}\left(i_{1}\right)}^{in}$, $\delta {\hat{a}}_{s_{2}\left(i_{2}\right)}^{out}=\sqrt{2\gamma_{2}}\delta {\hat{a}}_{s(i)}-\delta {\hat{a}}_{s_{2}\left(i_{2}\right)}^{in}$, the expressions for the output optical fields (${\hat{a}}_{s_{1}}^{out}$, ${\hat{a}}_{i_{1}}^{out}$, ${\hat{a}}_{s_{2}}^{out}$, ${\hat{a}}_{i_{2}}^{out}$) were obtained from the frequency domain, (${\hat{a}}_{s_{1}}^{out}$, ${\hat{a}}_{i_{1}}^{out})$ and (${\hat{a}}_{s_{2}}^{out}$, ${\hat{a}}_{i_{2}}^{out}$) were two pairs of the Einstein–Podolsky–Rosen (EPR) entangled states. Notably, for the NOPA operating in de-amplification, the coupled fields of the output signal (${\hat{a}}_{s_{1}}^{out}$, ${\hat{a}}_{s_{2}}^{out}$) and idler fields (${\hat{a}}_{i_{1}}^{out}$, ${\hat{a}}_{i_{2}}^{out}$) in the $+45^{\circ }$ polarizing direction were the amplitude quadrature squeezed states ${\hat{c}}_{2}$ (${\hat{c}}_{3}$); however, in the $-45^{\circ }$, they were the phase quadrature squeezed states ${\hat{c}}_{1}$ (${\hat{c}}_{4}$). Then, the output optical fields ${\hat{a}}_{s_{1}}^{out}$, ${\hat{a}}_{i_{1}}^{out}$, and ${\hat{a}}_{s_{2}}^{out}$, ${\hat{a}}_{i_{2}}^{out}$ were split by two half-wave plates ($HWP_{1}$, $HWP_{2}$) and two polarizing beam splitters ($PBS_{1}$, $PBS_{2}$), followed by superimposition. The superposed fields are expressed as shown below:

(2) $$\begin{array}{ccc} {\hat{c}}_{1} & = & \frac{1}{\sqrt{2}}\left({\hat{a}}_{s_{1}}^{out}-{\hat{a}}_{i_{1}}^{out}\right)\\ {\hat{c}}_{2} & = & \frac{1}{\sqrt{2}}\left({\hat{a}}_{s_{1}}^{out}+{\hat{a}}_{i_{1}}^{out}\right)\\ {\hat{c}}_{3} & = & \frac{1}{\sqrt{2}}\left({\hat{a}}_{s_{2}}^{out}+{\hat{a}}_{i_{2}}^{out}\right)\\ {\hat{c}}_{4} & = & \frac{1}{\sqrt{2}}\left({\hat{a}}_{s_{2}}^{out}-{\hat{a}}_{i_{2}}^{out}\right) \end{array}$$

Next, we consider how to obtain the spatially separated quadripartite GHZ state. First is to obtain fields ${\hat{c}}_{5}$ and ${\hat{c}}_{6}$, the superposed fields ${\hat{c}}_{2}$ and ${\hat{c}}_{3}$ were interfered on $BS_{1}$ (50/50) at a phase difference of π/2, as shown below:

(3) $$\begin{array}{ccc} {\hat{c}}_{5} & = & \frac{1}{\sqrt{2}}\left(\sqrt{\zeta_{2}}{\hat{c}}_{2}+\sqrt{1-\zeta_{2}}{\hat{a}}_{\nu 2}+i\sqrt{\zeta_{2}}{\hat{c}}_{3}+i\sqrt{1-\zeta_{2}}{\hat{a}}_{\nu 3}\right)\\ {\hat{c}}_{6} & = & \frac{1}{\sqrt{2}}\left(\sqrt{\zeta_{2}}{\hat{c}}_{2}+\sqrt{1-\zeta_{2}}{\hat{a}}_{\nu 2}-i\sqrt{\zeta_{2}}{\hat{c}}_{3}-i\sqrt{1-\zeta_{2}}{\hat{a}}_{\nu 3}\right) \end{array}$$

where $\zeta_{2}$ is the imperfect transmission efficiency of ${\hat{c}}_{2}$ (${\hat{c}}_{3}$).

Then, combining fields ${\hat{c}}_{1}$ and ${\hat{c}}_{5}$ on $BS_{2}$ (50/50), and ${\hat{c}}_{4}$ and ${\hat{c}}_{6}$ on CBS (50/50) with a phase difference of 0, four final optical fields ${\hat{d}}_{i}$ (i=1,2,3,4) were detected using balanced homodyne detectors BHD${}_{i}$ (i=1,2,3,4) with the detection efficiency η, as shown below:

(4) $$\begin{array}{ccc} {\hat{d}}_{1} & = & \frac{\sqrt{\eta }}{\sqrt{2}}\left(\sqrt{\zeta_{3}}{\hat{c}}_{5}+\sqrt{1-\zeta_{3}}{\hat{a}}_{\nu 5}-\sqrt{\zeta_{1}}{\hat{c}}_{1}-\sqrt{1-\zeta_{1}}{\hat{a}}_{\nu 1}\right)+\sqrt{1-\eta }{\hat{a}}_{\nu 7}\\ {\hat{d}}_{2} & = & \frac{\sqrt{\eta }}{\sqrt{2}}\left(\sqrt{\zeta_{3}}{\hat{c}}_{5}+\sqrt{1-\zeta_{3}}{\hat{a}}_{\nu 5}+\sqrt{\zeta_{1}}{\hat{c}}_{1}+\sqrt{1-\zeta_{1}}{\hat{a}}_{\nu 1}\right)+\sqrt{1-\eta }{\hat{a}}_{\nu 8}\\ {\hat{d}}_{3} & = & \frac{\sqrt{\eta }}{\sqrt{2}}\left(\sqrt{\zeta_{3}}{\hat{c}}_{6}+\sqrt{1-\zeta_{3}}{\hat{a}}_{\nu 6}+\sqrt{\zeta_{1}}{\hat{c}}_{4}+\sqrt{1-\zeta_{1}}{\hat{a}}_{\nu 4}\right)+\sqrt{1-\eta }{\hat{a}}_{\nu 9}\\ {\hat{d}}_{4} & = & \frac{\sqrt{\eta }}{\sqrt{2}}\left(\sqrt{\zeta_{3}}{\hat{c}}_{6}+\sqrt{1-\zeta_{3}}{\hat{a}}_{\nu 6}-\sqrt{\zeta_{1}}{\hat{c}}_{4}-\sqrt{1-\zeta_{1}}{\hat{a}}_{\nu 4}\right)+\sqrt{1-\eta }{\hat{a}}_{\nu 10} \end{array}$$

where $\zeta_{3}$ is the imperfect transmission efficiency of ${\hat{c}}_{5}$ (${\hat{c}}_{6}$), $\zeta_{1}$ is the imperfect transmission efficiency of ${\hat{c}}_{1}$ (${\hat{c}}_{4}$), and ${\hat{a}}_{\nu i}$ (i=1,2,……,10) are the vacuum noises caused by the imperfect transmission and detection efficiencies, respectively.

We subsequently calculated the correlation variances of the quadrature field component ${\hat{d}}_{i}$ for the GHZ state, followed by a characterization of the quadripartite GHZ state’s performance, employing the following inseparability criteria [44]:

(5) $$\begin{array}{ccc} V_{1}^{G} & = & \delta^{2}\left({\hat{Y}}_{d_{1}}-{\hat{Y}}_{d_{2}}\right)+\delta^{2}\left({\hat{X}}_{d_{1}}+{\hat{X}}_{d_{2}}+g_{3}{\hat{X}}_{d_{3}}+g_{4}{\hat{X}}_{d_{4}}\right)⩾6\\ V_{2}^{G} & = & \delta^{2}\left({\hat{Y}}_{d_{2}}-{\hat{Y}}_{d_{3}}\right)+\delta^{2}\left(g_{1}{\hat{X}}_{d_{1}}+{\hat{X}}_{d_{2}}+{\hat{X}}_{d_{3}}+g_{4}{\hat{X}}_{d_{4}}\right)⩾6\\ V_{3}^{G} & = & \delta^{2}\left({\hat{Y}}_{d_{3}}-{\hat{Y}}_{d_{4}}\right)+\delta^{2}\left(g_{1}{\hat{X}}_{d_{1}}+g_{2}{\hat{X}}_{d_{2}}+{\hat{X}}_{d_{3}}+{\hat{X}}_{d_{4}}\right)⩾6 \end{array}$$

where $g_{i}$ (i=1,2,3,4) are the adjustable measured gains; all of them were considered to be identical, as $g_{i}=g$.

When the combinations of correlation variances $V_{1}^{G}$, $V_{2}^{G}$, $V_{3}^{G}$ are simultaneously less than the quantum noise limit (QNL), the full inseparability quadripartite CV GHZ state is demonstrated. Hence, we also obtained equations for the correlation variance combinations in the GHZ state, as follows:

(6) $$\begin{array}{ccc} V_{1}^{G} & = & 4\kappa \eta \left[\frac{{\left(g-1\right)}^{2}\gamma_{2}\zeta_{2}\zeta_{3}}{{\left(-\kappa +\gamma \right)}^{2}+\tau^{2}\omega^{2}}-\frac{2\gamma_{1}\zeta_{1}+{\left(g+1\right)}^{2}\gamma_{1}\zeta_{2}\zeta_{3}}{{\left(\kappa +\gamma \right)}^{2}+\tau^{2}\omega^{2}}\right]+4+2g^{2}\\ & & +g\eta \zeta_{3}\left[2+\left(g-2\right)\zeta_{2}-2\sqrt{1-\zeta_{2}}\sqrt{1-\zeta_{3}}-g\zeta_{3}\right]\\ V_{2}^{G} & = & \frac{2{\left(g-1\right)}^{2}\kappa \eta \zeta_{1}{\left(\sqrt{\gamma_{1}}+\sqrt{\gamma_{2}}\right)}^{2}}{{\left(-\kappa +\gamma \right)}^{2}+\tau^{2}\omega^{2}}\\ & & -\frac{2\kappa \eta \zeta_{1}{\left(\sqrt{\gamma_{1}}-\sqrt{\gamma_{2}}\right)}^{2}+4\kappa \eta \zeta_{2}\zeta_{3}\left[{\left(1+g\right)}^{2}\gamma_{1}+\gamma_{2}\right]}{{\left(\kappa +\gamma \right)}^{2}+\tau^{2}\omega^{2}}\\ & & -\frac{1}{4}\eta \zeta_{3}g\left(2+g\right)\left(\zeta_{2}+\zeta_{3}+2\sqrt{1-\zeta_{2}}\sqrt{1-\zeta_{3}}-2\right)+4+2g^{2}\\ & & +\frac{1}{2}\eta \zeta_{3}\left(\zeta_{2}-\zeta_{3}\right)\\ V_{3}^{G} & = & \frac{4{\left(g-1\right)}^{2}\kappa \gamma_{2}\eta \zeta_{2}\zeta_{3}}{{\left(-\kappa +\gamma \right)}^{2}+\tau^{2}\omega^{2}}-\frac{8\kappa \gamma_{1}\eta \zeta_{1}+4{\left(g+1\right)}^{2}\kappa \gamma_{1}\eta \zeta_{2}\zeta_{3}}{{\left(\kappa +\gamma \right)}^{2}+\tau^{2}\omega^{2}}+4+2g^{2}\\ & & +\eta \zeta_{3}\left(\zeta_{2}-\zeta_{3}\right)-2g\eta \zeta_{3}\left(\zeta_{2}+\sqrt{1-\zeta_{2}}\sqrt{1-\zeta_{3}}-1\right) \end{array}$$

where ω is the analysis frequency.

Alternatively, this compact entanglement source could also produce the linear cluster state; hence, by just changing the relative phase between ${\hat{c}}_{4}$ and ${\hat{c}}_{6}$ to π/2 on CBS, we obtained the four final optical fields in the linear cluster state: ${\hat{d}}_{i}$ (i=1,2,3,4), which could be expressed by the transformation matrix $U_{L}$, as shown below:

(7) $$U_{L}=\left(\begin{array}{cccc} \sqrt{1-T_{1}^{\prime }} & \sqrt{\left(1-T_{0}^{\prime }\right)T_{1}^{\prime }} & i\sqrt{T_{0}^{\prime }T_{1}^{\prime }} & 0\\ i\sqrt{T_{1}^{\prime }} & -i\sqrt{\left(1-T_{0}^{\prime }\right)\left(1-T_{1}^{\prime }\right)} & \sqrt{T_{0}^{\prime }\left(1-T_{1}^{\prime }\right)} & 0\\ 0 & \sqrt{T_{0}^{\prime }\left(1-T_{2}^{\prime }\right)} & -i\sqrt{\left(1-T_{0}^{\prime }\right)\left(1-T_{2}^{\prime }\right)} & i\sqrt{T_{2}^{\prime }}\\ 0 & i\sqrt{T_{0}^{\prime }T_{2}^{\prime }} & \sqrt{\left(1-T_{0}^{\prime }\right)T_{2}^{\prime }} & \sqrt{1-T_{2}^{\prime }} \end{array}\right)$$

However, for the quadripartite CV linear cluster state, the corresponding inseparability criteria are as follows [45]:

(8) $$\begin{array}{ccc} V_{1}^{C} & = & \delta^{2}\left({\hat{Y}}_{d_{1}}-{\hat{X}}_{d_{2}}\right)+\delta^{2}\left({\hat{Y}}_{d_{2}}-{\hat{X}}_{d_{1}}-{\hat{X}}_{d_{3}}\right)⩾5\\ V_{2}^{C} & = & \delta^{2}\left({\hat{Y}}_{d_{2}}-{\hat{X}}_{d_{1}}-{\hat{X}}_{d_{3}}\right)+\delta^{2}\left({\hat{Y}}_{d_{3}}-{\hat{X}}_{d_{2}}-{\hat{X}}_{d_{4}}\right)⩾6\\ V_{3}^{C} & = & \delta^{2}\left({\hat{Y}}_{d_{3}}-{\hat{X}}_{d_{4}}\right)+\delta^{2}\left({\hat{Y}}_{d_{3}}-{\hat{X}}_{d_{2}}-{\hat{X}}_{d_{4}}\right)⩾5 \end{array}$$

In this case, when the three above equalities of the correlation variance combinations $V_{1}^{C}$, $V_{2}^{C}$, $V_{3}^{C}$ were simultaneously less than QNL, the obtained four optical fields ${\hat{d}}_{i}\;(i=1,2,3,4$) were in a full inseparable linear cluster state; however, when $T_{0}^{\prime }=\frac{1}{5}$ and $T_{1}^{\prime }=T_{2}^{\prime }=\frac{1}{2}$, the quadripartite linear cluster state was generated. In this way, the calculated correlation variance combinations of fields ${\hat{d}}_{i}$ for a linear cluster state were finally obtained, as shown below:

(9) $$\begin{array}{ccc} V_{1}^{C} & = & 5-\frac{2(4\gamma_{1}\zeta_{1}+\gamma_{2}\zeta_{1}+5\gamma_{1}\zeta_{2}\zeta_{3})\kappa \eta }{{\left(\kappa +\gamma \right)}^{2}+\tau^{2}\omega^{2}}\\ V_{2}^{C} & = & 6-\frac{2\kappa \left(\gamma_{1}+\gamma_{2}\right)\eta \left(\zeta_{1}+5\zeta_{2}\zeta_{3}\right)}{{\left(\kappa +\gamma \right)}^{2}+\tau^{2}\omega^{2}}\\ V_{3}^{C} & = & 5-\frac{2(\gamma_{1}\zeta_{1}+4\gamma_{2}\zeta_{1}+5\gamma_{2}\zeta_{2}\zeta_{3})\kappa \eta }{{\left(\kappa +\gamma \right)}^{2}+\tau^{2}\omega^{2}} \end{array}$$

## 3 Simulation results

**Fig. 2:**
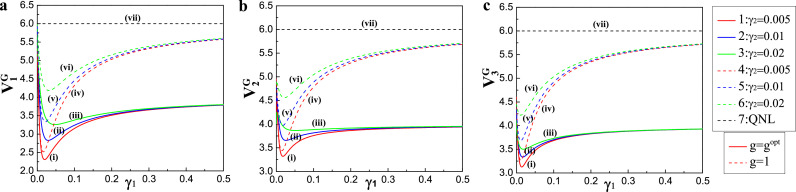
**Schematic showing on the dependence of the correlation variance combinations for the GHZ state on the coupling rates**$\gamma_{1}$**of the output coupler**$M_{3}$. (a) $V_{1}^{G}$, (b) $V_{2}^{G}$, (c) $V_{3}^{G}$. The traces (vii) were QNLs, traces (i) ((iv)), (ii) ((v)), and (iii) ((vi)) were the calculated combinations of the correlation variances when the coupling rates $\gamma_{2}$ were selected as 0.005, 0.01, and 0.02, respectively. The solid and dashed lines correspond to $g=g^{opt}$ and g=1, respectively.

This study investigated the correlation variances of spatially separated quadripartite GHZ and linear cluster states. Fig. 2 demonstrates the dependence of the correlation variance combinations $V_{1}^{G}$, $V_{2}^{G}$, and $V_{3}^{G}$ on the coupling rate $\gamma_{1}$ of the output coupler $M_{3}$. Fig. 2a–c corresponds to $V_{1}^{G}$, $V_{2}^{G}$, and $V_{3}^{G}$, respectively. Investigations revealed that the parameter values were experimentally reachable, providing the direct references. In our scheme, the parameters were taken as follows: the analysis frequency, ω=2.0 MHz; the length of the NOPA, l=560 mm; the parametric coupling constant of the crystal, κ=0.01; the corresponding decay rate of intracavity loss, $\gamma_{3}=0.001$; the transmission efficiency of ${\hat{c}}_{2}$ (${\hat{c}}_{3}$) and ${\hat{c}}_{5}$ (${\hat{c}}_{6}$), $\zeta_{2}=\zeta_{3}=0.98$; the transmission efficiency of ${\hat{c}}_{1}$ (${\hat{c}}_{4}$), $\zeta_{1}=0.96$; and the detection efficiency, η=0.98. Next, we optimized the adjustable measured gain to $g^{opt}$ (the solid and dashed lines correspond to $g=g^{opt}$ and g=1, respectively). It was evident that the correlation variance combinations of the optimal gain $g^{opt}$ were smaller than those of the unit gain. Notably, traces (vii) were QNLs, and traces (i) ((iv)), (ii) ((v)), and (iii) ((vi)) were the calculated correlation variance combinations when the coupling rate $\gamma_{2}$ was selected as 0.005, 0.01, and 0.02, respectively. Investigations also revealed that the correlation variance combinations were affected by the coupling rates $\gamma_{1}$ and $\gamma_{2}$ of the output couplers ($M_{3}$ and $M_{4}$). When $\gamma_{2}>0$, the quadripartite GHZ state was generated. As the coupling rate $\gamma_{2}$ became larger, the minimum values of the correlation variance combinations $V_{1}^{G}$, $V_{2}^{G}$, and $V_{3}^{G}$ increased gradually, indicating the existence of an optimal coupling rate $\gamma_{1}$ for minimizing the correlation variance combinations. Furthermore, we observed that all correlation variance combinations were below the QNL in a broad range, suggesting that the compact and deterministic entanglement source could be efficient for the four users.

Whereas, when taking $\gamma_{2}=0.005$, the minimum values of the correlation variance combinations $V_{1}^{G}$, $V_{2}^{G}$, and $V_{3}^{G}$ were close to the minimum values, with the corresponding optimal values of $\gamma_{1}$ being about 0.01. This result shows that all three inequalities in Eq. 5 were violated, thus demonstrating the spatially separated quadripartite CV GHZ state of the four optical fields.

**Fig. 3:**
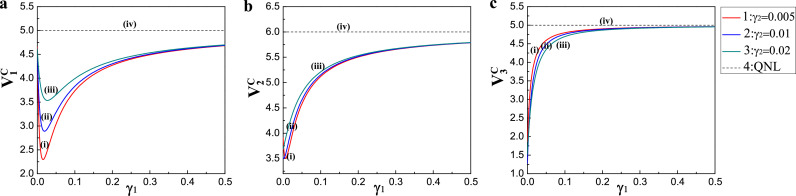
**Schematic showing on the functions of the correlation variance combinations for a linear cluster state on the coupling rates**$\gamma_{1}$**of the output coupler**$M_{3}$. (a) $V_{1}^{C}$, (b) $V_{2}^{C}$, (c) $V_{3}^{C}$. Traces (iv) are QNLs, and traces (i), (ii), and (iii) show the correlation variance combinations with the coupling rate $\gamma_{2}=0.005,0.01,0.02$, respectively.

We subsequently analyzed the linear cluster state. The functions of the correlation variance combinations $V_{1}^{C}$, $V_{2}^{C}$, and $V_{3}^{C}$ versus the coupling rate $\gamma_{1}$ of the output coupler $M_{3}$ are shown in Fig. 3. All the parameter values were the same as those in Fig. 2. Note that Fig. 3a–c corresponds to $V_{1}^{C}$, $V_{2}^{C}$, and $V_{3}^{C}$, respectively. Traces (iv) are QNLs, and traces (i), (ii), and (iii) show the correlation variance combinations at various coupling rates $\gamma_{2}=0.005,0.01,0.02$, respectively. From the theoretical simulation results for the linear cluster state, when $\gamma_{1}$ and $\gamma_{2}$ were 0.01 and 0.005, respectively, the values of $V_{1}^{C}$, $V_{2}^{C}$, and $V_{3}^{C}$ were close to the minimum values. Thus, all the inequalities in Eq. 8 were simultaneously violated, verifying the full inseparability of the produced linear cluster state [50].

Based on the above simulation results, we considered some parameters as follows: the analysis frequency, ω=2.0 MHz; the length of the NOPA, l=560 mm; the parametric coupling constant of the crystal, κ=0.01; the coupling rate of the output coupler $M_{3}$, $\gamma_{1}=0.01$; the coupling rate of the output coupler $M_{4}$, $\gamma_{2}=0.005$; the corresponding decay rate of intracavity loss, $\gamma_{3}=0.001$; the transmission efficiency of ${\hat{c}}_{2}$ (${\hat{c}}_{3}$) and ${\hat{c}}_{5}$ (${\hat{c}}_{6}$), $\zeta_{2}=\zeta_{3}=0.98$; the transmission efficiency of ${\hat{c}}_{1}$ (${\hat{c}}_{4}$), $\zeta_{1}=0.96$; and the detection efficiency, η=0.98. Notably, the values of $V_{1}^{G}$, $V_{2}^{G}$, $V_{3}^{G}$, $V_{1}^{C}$, $V_{2}^{C}$, and $V_{3}^{C}$ were below the boundary values of the inseparability criteria; therefore, this compact entanglement source flexibly produced the quadripartite CV GHZ and linear cluster states by controlling the phase difference of CBS.

The threshold power of NOPA with two output ports is higher than that of the NOPAs or DOPAs with one output port under the same parameters, but the required threshold power of this quantum source is low enough for practical applications; nevertheless, the threshold power of NOPA depends on the transmissivity and intracavity loss. Hence, for two-ports NOPA in this study, the transmissivities of output ports and intracavity loss were small, which guaranteed enough threshold power.

Even though the entangled degree of quadripartite entanglement generated from one NOPA with two output ports is lower than that generated from two (four) traditional NOPAs (DOPAs), the cost for quantum resources is minimum. For a fixed gain of 0.8 in the parametric process with the coupling rate of the output coupler $\gamma_{1}=0.01$, the transmission efficiency ζ=0.98, the detection efficiency η=0.98, and the squeezed degree of the DOPA or the NOPA with one output port was 7 dB; four DOPAs or two NOPAs were required for generating quadripartite entangled optical feilds. But, our proposed scheme can, in principle, still make the source compact. Hence, under the above conditions, quadripartite entangled optical fields with an entangled degree of 2.5 dB were generated using the two ports NOPA. The resultant CV GHZ and linear cluster states could potentially be applied in quantum-enhanced information science, specifically in quantum secret sharing, controlled quantum teleportation networks, and quantum entangled atomic ensemble networks.

## 4 Conclusion

This study proposed a compact and feasible quantum source that could deterministically entangle four spatially separated optical fields. In our protocol, we needed only one NOPA and an optical beam splitter network. Based on the criteria, both the GHZ and the cluster states were flexibly generated from the proposed compact source; they could potentially be applied to quantum-enhanced information science, specifically to quantum secret sharing [11], controlled quantum teleportation networks [51], and quantum-entangled atomic ensemble networks [52], [53], [54]. But, we encountered a major challenge in this scheme: constructing a quantum network with more users; nevertheless, this issue was resolved by employing more NOPAs. For example, the entanglement among eight-partite was obtained using two NOPAs with two output ports. The other challenge was that significant improvement in the entangled degree of the multipartite entangled optical fields was still needed for our method. To this end, we used the coherent feedforward control to improve the entangled degree [55], [56]. Besides, the entangled degree mainly depends on the transmissivity of output port and intracavity loss of the NOPA. On the one hand, it is an effective approach to optimize the intracavity coating quality for better entangled degrees. On the other hand, it is also possible to increase the transmissivity of the output port, thereby improving both the entangled degree and the threshold. Consequently, a solid-state laser was developed, serving as the foundation for low-noise and high-power lasers, which guarantees a high enough pump power for practical applications [57]. Hence, based on the above techniques, the entangled degree of multipartite entangled optical fields was further improved, indicating that our protocol paves a feasible way for the practical applications of a large-scale quantum network.

## Declaration of competing interest

The authors declare that they have no conflicts of interest in this work.
